# RETRACTED ARTICLE: Evaluating the neuroprotective effect of *Spirulina platensis*–loaded niosomes against Alzheimer’s disease induced in rats

**DOI:** 10.1007/s13346-023-01301-2

**Published:** 2023-02-15

**Authors:** Asmaa K. Abdelghany, Amr Gamal, Ahmed Abdel-Wahab, Abdel-Razik H. Abdel-Razik, Salma.I. El-Samannoudy, Marwa A. Ibrahim, Walid Hamdy Hassan, Fatma I. Abo El-Ela

**Affiliations:** 1 Animal and Poultry Management and Wealth Development Department, Faculty of Veterinary Medicine, Beni-Suef University, Beni-Suef, 62511 Egypt; 2 Department of Pharmaceutics and Industrial Pharmacy, Faculty of Pharmacy, Beni-Suef University, Beni-Suef, Egypt; 3 Department of Physiology, Faculty of Veterinary Medicine, Minia University, El-Minia, Egypt; 4 Department of Histopathology, Faculty of Veterinary Medicine, Beni-Suef University, Beni-Suef, 62511 Egypt; 5 Physiology Department Faculty of Veterinary Medicine, Cairo University, Giza, Egypt; 6 Department of Biochemistry and Molecular Biology, Faculty of Veterinary Medicine, Cairo University, Giza, 12211 Egypt; 7 Department of Microbiology Mycology and Immunology, Faculty of Veterinary Medicine, Beni-Suef University, Beni-Suef, 62511 Egypt; 8 Department of Pharmacology, Faculty of Veterinary Medicine, Beni-Suef University, Beni-Suef, 62511 Egypt

The Editor-in-Chief has retracted this article. After publication, concerns were raised regarding image overlap in the presented figures. Specifically:

Fig. 1 TEM images appear to contain multiple highly similar areas.

Fig. 8 a4 and b1 appear to contain an overlapping area.

Fig. 9 d4 image appears to contain two highly similar areas.

The authors have stated that the images in Fig. 1 originated from the same source image. They have also provided raw data to address these concerns; however, the same similarities were found in the raw data files by the publisher. The Editor-in-Chief therefore no longer has confidence in the presented data.

None of the authors agree to this retraction.

The online version of this article contains the full text of the retracted article as Supplementary Information.

## Supplementary Information

Below is the link to the electronic supplementary material.

Former article version (PDF 4143 KB)

