# Supplementary material for: RETRACTED ARTICLE: Evaluating the neuroprotective effect of Spirulina platensis–loaded niosomes against Alzheimer’s disease induced in rats
Source: Drug Deliv Transl Res. 2023 Feb 15;13(10):2690. doi: 10.1007/s13346-023-01301-2 (PMC10468951; doi:10.1007/s13346-023-01301-2)
Supplement: Supplementary file 1 — Former article version (PDF 4143 KB) [file 13346_2023_1301_MOESM1_ESM.pdf]

Drug Delivery and Translational Research
https://doi.org/10.1007/s13346-023-01301-2

ORIGINAL ARTICLE

Check for updates

Evaluating the neuroprotective effect of *Spirulina platensis*–loaded niosomes against Alzheimer's disease induced in rats

Asmaa K. Abdelghany[1] · Amr Gamal[2] · Ahmed Abdel-Wahab[3] · Abdel-Razik H. Abdel-Razik[4] · Salma.I. El-Samannoudy[5] · Marwa A. Ibrahim[6] · Walid Hamdy Hassan[7] · Fatma I. Abo El-Ela[8]

Accepted: 18 January 2023
© The Author(s) 2023

RETRACTED ARTICLE

Abstract

Alzheimer's disease (AD) is a progressive neurodegenerative disease that is characterized by memory loss, changes in behavior, and avoidance of social bonds. Aluminum is one of the main risk factors in the development of AD. *Spirulina platensis* (SP) is a microalga that improves motor and cognitive skills and prevents cerebral endothelial damage. SP could be delivered in a more controlled and targeted manner using nanoparticles like niosomes. The purpose of this research was to develop a SP-loaded niosome (SPLN) formulation as a drug delivery system to investigate the effectiveness and toxicity of SP as an AD therapy using the $AlCl_3$-induced AD rat model. A niosomal formulation that consists of Tween 60, cholesterol, and dihexadecyl phosphate in a molar ratio of 1:2:0.1 was chosen as an optimum formulation. AD was induced in rats by orally administering $AlCl_3$. Compared with the $AlCl_3$ control, the group treated with the SPLN formulation showed enhancement of the recognition and working memories by increasing the difference score, the discrimination ratio, and the spontaneous alternation behavior. Additionally, it revealed a significant increase in AchE genes, restored the reduced brain neurotransmitters, and improved brain oxidative status. In conclusion, SPLN formulation could be considered an effective AD therapy.

Keywords Alzheimer's disease · Neuroprotective · *Spirulina platensis* · Niosomes · Gene expression

Introduction

Alzheimer's disease (AD) is a progressive neurodegenerative disease that causes amyloid and tangles to accumulate in the brain [1, 2]. To date, AD has affected over 25 million people all over the globe [3, 4]. It is

✉ Fatma I. Abo El-Ela
Fatma.aboel3la@vet.bsu.edu.eg; fa.[illegible]@yahoo.com

Asmaa K. Abdelghany
Asmaa.kamal@vet.bs[illegible]du.eg

Amr Gamal
Amr_g@pharm.[illegible].edu.eg

Ahmed Abdel-Wahab
ahmed.abdelwahab@m[illegible].edu.eg

Abdel-Razik H. Abdel-Razik
a[illegible]lrazak[illegible]an@vet.bsu.edu.eg

Salma.I. El-Samannoudy
[illegible]ma.elsamanoody@vet.cu.edu.eg

Marwa A. Ibrahim
Marwaibrahim@cu.edu.eg

Walid Hamdy Hassan
walidhamdyhassan@yahoo.com

[1] Animal and Poultry Management and Wealth Development Department, Faculty of Veterinary Medicine, Beni-Suef University, Beni-Suef 62511, Egypt

[2] Department of Pharmaceutics and Industrial Pharmacy, Faculty of Pharmacy, Beni-Suef University, Beni-Suef, Egypt

[3] Department of Physiology, Faculty of Veterinary Medicine, Minia University, El-Minia, Egypt

[4] Department of Histopathology, Faculty of Veterinary Medicine, Beni-Suef University, Beni-Suef 62511, Egypt

[5] Physiology Department Faculty of Veterinary Medicine, Cairo University, Giza, Egypt

[6] Department of Biochemistry and Molecular Biology, Faculty of Veterinary Medicine, Cairo University, Giza 12211, Egypt

[7] Department of Microbiology Mycology and Immunology, Faculty of Veterinary Medicine, Beni-Suef University, Beni-Suef 62511, Egypt

[8] Department of Pharmacology, Faculty of Veterinary Medicine, Beni-Suef University, Beni-Suef 62511, Egypt

RETRACTED ARTICLE

characterized by memory loss, changes in mood and behavior, and avoidance of social bonds [5]. One of the most significant heavy metals in the development and spread of AD is aluminum (Al), which is found in a variety of commercially created products [6, 7]. Al has a direct impact on the nervous system's multiple metabolic cascades [7]. It upregulates the expression of AD-related pathogenic genes like interleukin-1, interleukin-6, tumor necrosis factor, acetylcholine esterase, and monoamine oxidase [6, 7]. In the molecular mechanism of AD pathogenesis, Al causes neurotoxicity and amyloid protein oligomerization [8].

Spirulina platensis (SP) is a type of cyanobacteria that thrives in warm, alkaline freshwater lakes [9]. Several studies have shown that SP has potential benefits for brain health [9–11]. It has antioxidant, anti-inflammatory, and neuroprotective properties [9, 10, 12]. It mitigates mental fatigue via preventing endothelial damage to cerebral blood vessels [10, 11]. Additionally, it improves motor, language, and cognitive skills [13]. Drugs could be delivered in a more controlled and specific manner using nanoparticles like liposomes and niosomes [14–16]. The drug's bioavailability, efficiency, and selectivity are all enhanced by the nanoparticle delivery system [14, 15, 17]. Since phospholipids are easily oxidized and hydrolyzed, niosomes are superior to liposomes [14, 15, 18]. Niosomes are targeted drug delivery systems that use non-ionic surfactants and cholesterol to increase the delivery of water-soluble drugs such as SP [14, 15, [illegible], 19]. Niosomes have been found to improve dr[illegible] stability, decrease toxicity, increase circulation time, and [illegible]hance target-site uptake [14, 15, 17, 20]. The purpose [illegible] this research was to develop an SP-loade[illegible] niosome (SPLN) formulation as a drug delivery system [illegible] investigate the effectiveness and toxicity of S[illegible] an AD therapy using the $AlCl_3$-induced AD rat model. D[illegible]nt SPLN formulations were prepared a[illegible] evaluated to choose the optimum formulation for [illegible] studies such as the efficacy and toxicity of SPLN in [illegible]mparison to oral SP.

Materials and methods

Materials

Tw[illegible] 60, cholesterol, dihexadecyl phosphate, chloroform, [illegible]nd methanol were purchased from Agitech Pharmaceutical Company (Cairo, Egypt). Aluminum chloride anhydrous ($AlCl_3$) was imported from an Indian central drug store. *Spirulina platensis* was obtained as a green colored pure powder from the Amoun Vet. Company (AVC) for pharmaceutical drugs synthesis, Cairo, Egypt.

Preparation and in vitro characterization of Spirulina-loaded niosome formulation

Preparation of Spirulina-loaded niosome formulation

The thin-film hydration technique was used to prepare *Spirulina*-loaded niosomes (SPLN) [15]. The calculated amounts of Tween 60, cholesterol, and dihexadecyl phosphate (DDP) were dissolved in a solution (10 ml) of chloroform and methanol (3:1), with a molar ratio of 1:2:0.1. Next, th[illegible] solution was poured into a flask and evaporated at 100 rp[illegible] under vacuum using a Stuart rotary evaporator ([illegible]E300, UK). SP hydrated the film at 60 °C for 2h [illegible] dissolved in 10 ml of phosphate buffer (PB). The SPLN formulation was ultrasonicated for 30 min using a S[illegible]ix (IL, USA). Isolation of SPLN pellets required 1h of [illegible] at 15,000 rpm in a centrifuge (SIGMA, [illegible]). PB (10 ml) was added to SPLN pellets and [illegible] at 4 °C.

Morphology of SPLN formulation

The form[illegible]tion and [illegible] of SPLN vesicles were studied with transmiss[illegible]tron microscopy (Carl Zeiss, Germany) [21]. On a carbon-coated copper grid, 20 µl of the SPLN [illegible]mulation were deposited, colored with phosphotungstic dye, [illegible]nd allowed to dry.

Particle size and zeta potential determination

The dispersion, homogeneity, distribution, and, ultimately, the ability to target the particles are all influenced by their size and polydispersity index (PDI) [17]. Determining the zeta potential of the SPLN formulation allowed for an assessment of its electrostatic charge, surface characteristics, and stability [19, 22]. In order to measure the particle size, PDI, and zeta potential in three replicates, we diluted 1 ml of each SPLN formulation with 9 ml of distilled water and used dynamic light scattering (DLS, Germany) to analyze the results [21].

In vivo characterization of Spirulina-loaded niosome formulation

Alzheimer's disease induction

In this study, we used 40 male Wistar rats weighing between 120 and 150 g. Animals were housed in a well-ventilated room in plastic shoebox cages (70 × 35 × 20 cm) and allowed to accommodate for 7 days before starting the experiment. There was an unlimited supply of clean, fresh water and commercially prepared meals. The temperature range was 21–24.5 °C, and the range of relative humidity was 46–60%.

The lighting system was maintained using a reversed 12-h light–dark cycle. To make aluminum chloride, the powder was mixed with sterile water and stored in a dark bottle until it was ready to be taken orally. Alzheimer's disease was artificially induced by orally administering $AlCl_3$ (100 mg/kg) to each rat [23, 24]. All experiments in this work were performed in in accordance with the rules of the local ethical committee IACUC at the Faculty of Pharmacy, Beni-Suef University. The ethical committee approval number to the study is IACUC, BSU, (022-366).

Experimental design

The rats were split up into four groups of ten at random, as detailed below:

A: control negative group; a daily oral gavage of distilled water was used on each rat (neither $AlCl_3$ nor treatments were obtained).

B: $AlCl_3$ group (control positive group); a daily oral gavage of $AlCl_3$ (100 mg/kg) was used on each rat for 4 weeks.

C: $AlCl_3$/SP group; a daily oral gavage of SP (300 mg/kg b.wt) was used on each rat before $AlCl_3$ administration by 1 h [25].

D: $AlCl_3$/SPLN group; a daily oral gavage of SPLN (300 mg/kg b.wt) was used on each rat before $AlCl_3$ administration by 1 h.

Behavioral tests

Deficits in learning and memory in rats were evaluated using the novel object recognition (NOR) test. It relies on the rat's innate propensity to explore and investigate novel objects rather than rely on cues from previously encountered objects [26]. During the test days, a digital camera was utilized to record the computed parameters. The first part of the test (the familiarization phase) involved placing two known objects in the middle of the arena and leaving the rats there for 10 min. In the second phase (the test phase), which was conducted after 24 h, each rat spent 5 min with one familiar object and one novel object. All of the testing equipment, procedures, and calculation equations were carried out as per the detailed instructions given here [26–28].

To evaluate participants' spatial short-term working memory, we used the Y-maze to record their impromptu responses in each arm of the maze [29]. All of the testing equipment, procedures, and calculation equations were carried out as per the detailed instructions given here [30–32]. Throughout the test days, the estimated parameters were videotaped using a digital camera for recording. Each rat was put at the beginning of the (A) arm and left there for 8 min. The other two arms were labeled (B) and (C), respectively, as a result of calculating the order of arm entries in overlapping triplet sets (i.e., ABCCBAABC).

After the behavioral tests are finished, an intraperitoneal (IP) injection of a mixture (0.1 mg/100 g) of ketamine (90 mg/kg) and xylazine (5 mg/kg) at a ratio of 1:1 was administered to each rat for anesthesia. Rats were euthanized via cervical dislocation, and their brains were removed. The brain was dissected into three samples. For the purpose of determining oxidative stress markers, the first sample was homogenized in 5 ml of phosphate-buffered saline for 20 min using a homogenizer (Yellow line DI 18 basic, Deutschland, Germany), and the supernatant was collected. The second sample was placed at – 80 °C in a collection tube of saline, RNase, and protease inhibitors (Promega Corporation, USA) to be used in the determination of gene expression. For the histological analysis, the third sample was washed in saline and then submerged in Bouin's solution.

Determination of oxidative stress markers

Malondialdehyde (MDA) and total antioxidant capacity (TAC) levels in the brain were evaluated in tissue homogenates using particular rat colorimetric test kits [33]. The MDA kit was purchased from Egypt's Biodiagnostic Company (Catalogue Number: MD [illegible]29). The test is based on a 30-min reaction between thiobarbituric acid (TBA) and MDA at 95 °C in an acidic medium. As a result of this reaction, the thiobarbituric acid reactive product was produced. The resulting colored substance's absorbance was measured at 534 nm. The QuantiChrom™ Antioxidant Assay Kit was used to determine TAC levels (Catalogue Number: DTAC-100, Hayward, CA 94,545, USA). TAC is measured using Bio-Assay Systems' enhanced assay, which reduces Cu^{2+} to Cu^{+} using an antioxidant. Cu^{+} reacts with a dye reagent to produce a colorful product. The TAC of the sample is proportional to the color intensity at 570 nm.

Determination of acetylcholinesterase activity, brain monoamines, and acetylcholine levels

The levels of acetylcholinesterase (AchE) enzyme activity in brain homogenates were measured using a commercially available colorimetric assay kit (BioVision Co., Milpitas, CA, USA). Acetylcholine was determined using HPLC [34]. In 75% aqueous HPLC grade methanol (10% w/v), we weighed and homogenized each piece of brain tissue then reconstituted to 1 to 1 volume [35]. After a 10-min spin at 4000 rpm, the homogenate's supernatant was frozen at – 80 °C for later use. To detect monoamines in the brain, we used the HPLC technique reported by Pagel et al. [36]. The HPLC setup included a quaternary pump, column oven, Rheodyne injector, 20-μl loop, and UV–Vis-NIR variable wavelength detector. The chromatogram and report were generated by a Chemstation-purchased data acquisition application. Solid-phase extraction Chromabond column NH_2 phase cat. No.730031 was used

Table 1 The primer sets of the assessed genes

Gene	Forward primer	Reverse primer	Product	Accession no
Bax	CACGTCTGCGGGGAGTCAC	TTCTTGGTGGATGCGTCCTG	248	NM_017059.2
Bcl-2	TCGCGACTTTGCAGAGATGT	CAATCCTCCCCCAGTTCACC	116	NM_016993.2
AchE	AGG ACG AGG GCT CCT ACT TT	CAT GGC ATC TCT CAG GTG GG	200	NM_172009.1
Mao	GTG CCT GGT CTG CTC AAG AT	GGC CCA AAC CAT AGG CTG TA	168	NM_033653.1
ACTB	CCGCGAGTACAACCTTCTTG	CAGTTGGTGACAATGCCGTG	297	NM_031144.3

on the sample right away to remove all of the lipids and trace elements. Following this, the material was injected straight into an AQUA 150×5 mm C18 column from Phenomenex, USA, with a mobile phase composed of 20 mM potassium phosphate (pH 2.5) at a flow rate of 1.5 ml/min and a UV of 270 nm. After only 10 min, noradrenaline, dopamine, DOPAC, and serotonin were isolated. The ensuing chromatogram showed where each monoamine was located and how concentrated it was relative to the standard, allowing for an accurate calculation of how much of each monoamine was present in the brain tissue.

Gene expression

We used an RNeasy Mini Kit (Qiagen Cato./ID 74,104) to extract total RNA from brain tissue. SuperScript Reverse Transcriptases were used for first-strand cDNA synthesis [37]. For quantitative PCR, an ABI Prism StepOnePlus real-time PCR system with PowerTrack™ SYBR Green Master Mix Applied Biosystems™ (Applied Biosystems) was used [38]. The primer sets of the examined genes are compiled in Table 1. Following normalization of the target mRNA expression to ACTB, the following was determined:

ΔCt = Ct (gene of interest) − Ct (housekeeping gene);

ΔΔCt = ΔCt (treated sample) − ΔCt (untreated sample).

Histopathological examination

After being submerged in Bouin's solution, samples of brain tissue were dehydrated in progressively stronger concentrations of ethyl alcohol, cleared in xylol, impregnated with soft paraffin, and finally embedded in hard paraffin. Sections of 5–7 μm were cut and mounted on clear and dry glass slides. Histopathological analysis was performed using a LEICA (DFC290 HD system digital camera, Heerbrugg, Switzerland) attached to a light microscope, and the obtained slides were stained with hematoxylin and eosin (H&E) and Bielschowsky's silver stain [39]. The formation of amyloid plaques around neurons in the AD were observed by Congo red stain [40].

Statistical analysis

One-way analysis of variance (ANOVA) and the Tukey test were used to analyze the data using SPSS version 22. The Tukey test is a post hoc test commonly used to assess the significance of differences between pairs of group means [41]. It was used to determine whether the treatment group significantly differed from the control group or not. The data were presented as mean standard error of the mean, and they were statistically significant at the $P < 0.05$ level.

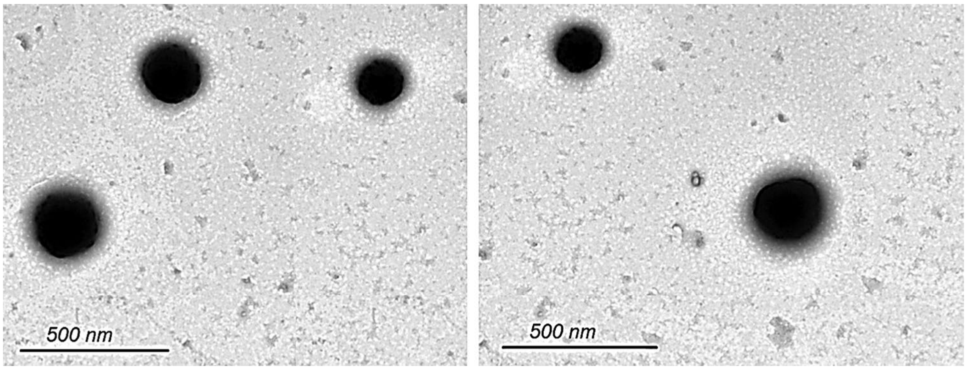

Fig. 1 Transmission electron microscopy of SPLN formulation

RETRACTED ARTICLE

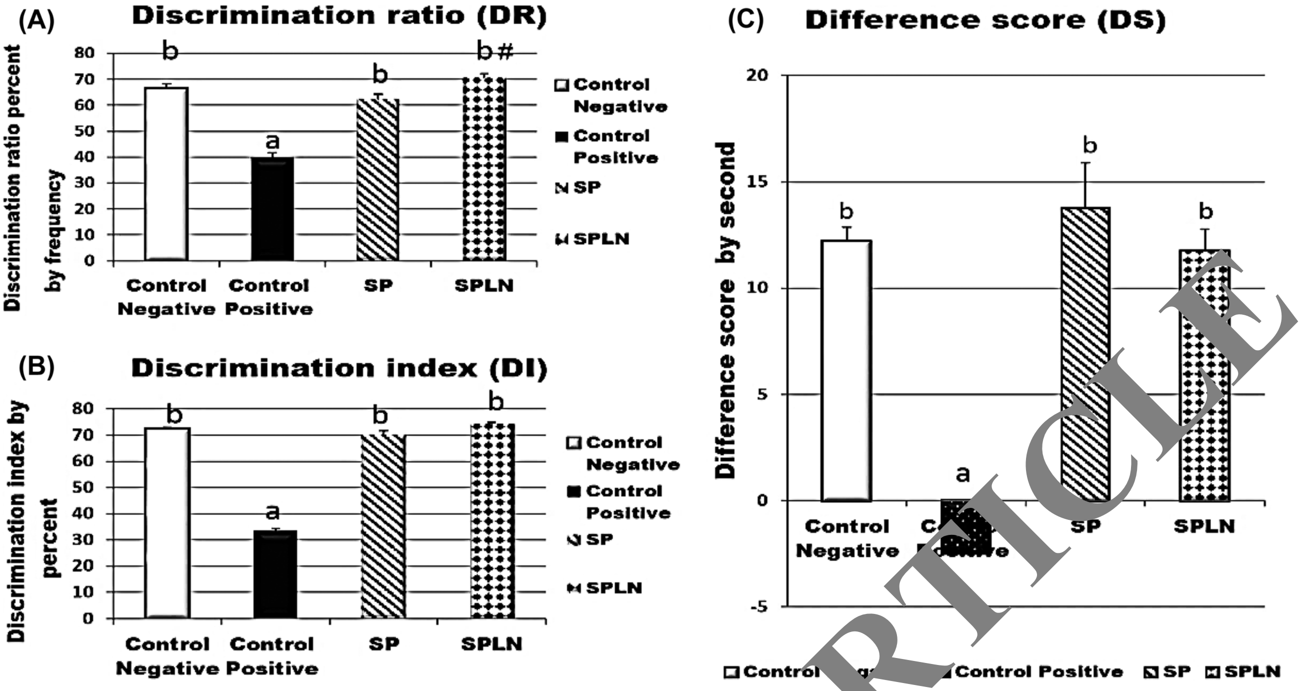

Fig. 2 Effect of SPLN formulation on recognition memory (NOR test). Results are expressed as means ± SE; The different superscript indicates significant difference at $P < 0.01$ and number sign indicated a significa[illegible]ference between SP and SPLN formulation at level $P < 0.05$ usi[illegible]ne-[illegible]ay ANOVA followed by Tukey post hoc test

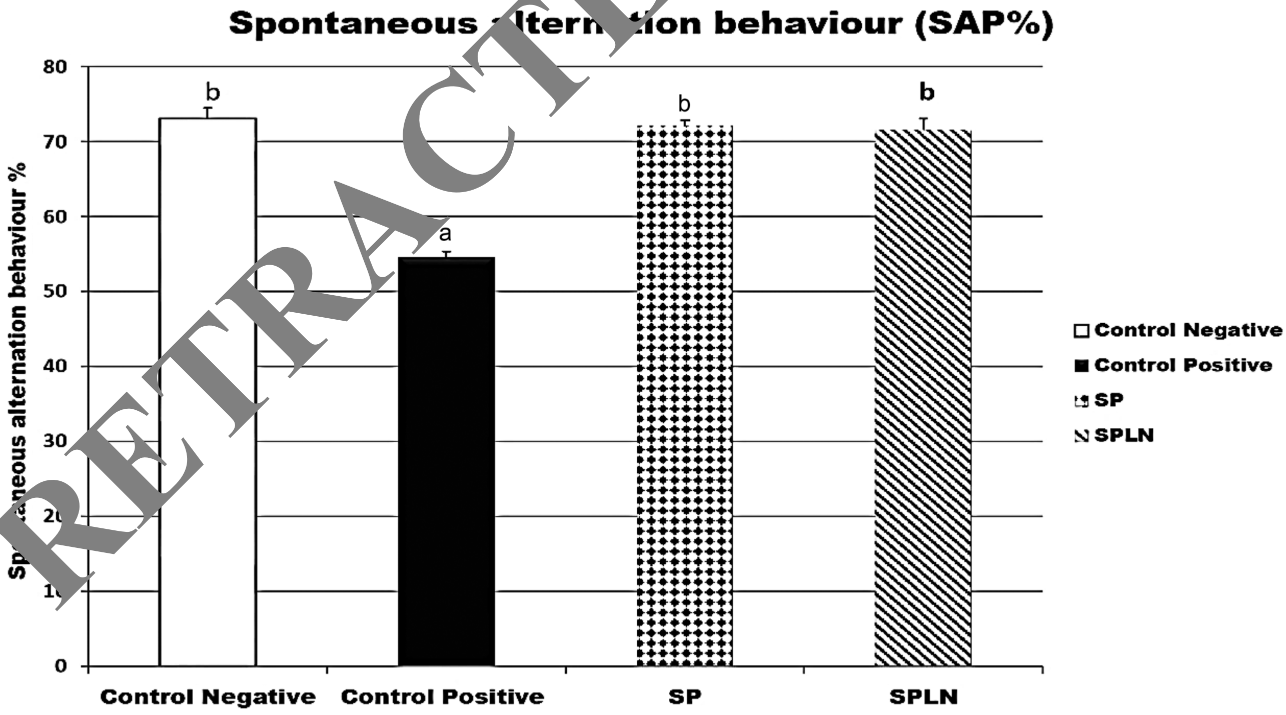

Fig. 3 Effect of SPLN formulation on spontaneous alternation behavior (Y-maze test). Results are expressed as means ± SE. The different superscript indicates significant difference at $P < 0.01$ using one-way ANOVA followed by Tukey post hoc test

RETRACTED ARTICLE

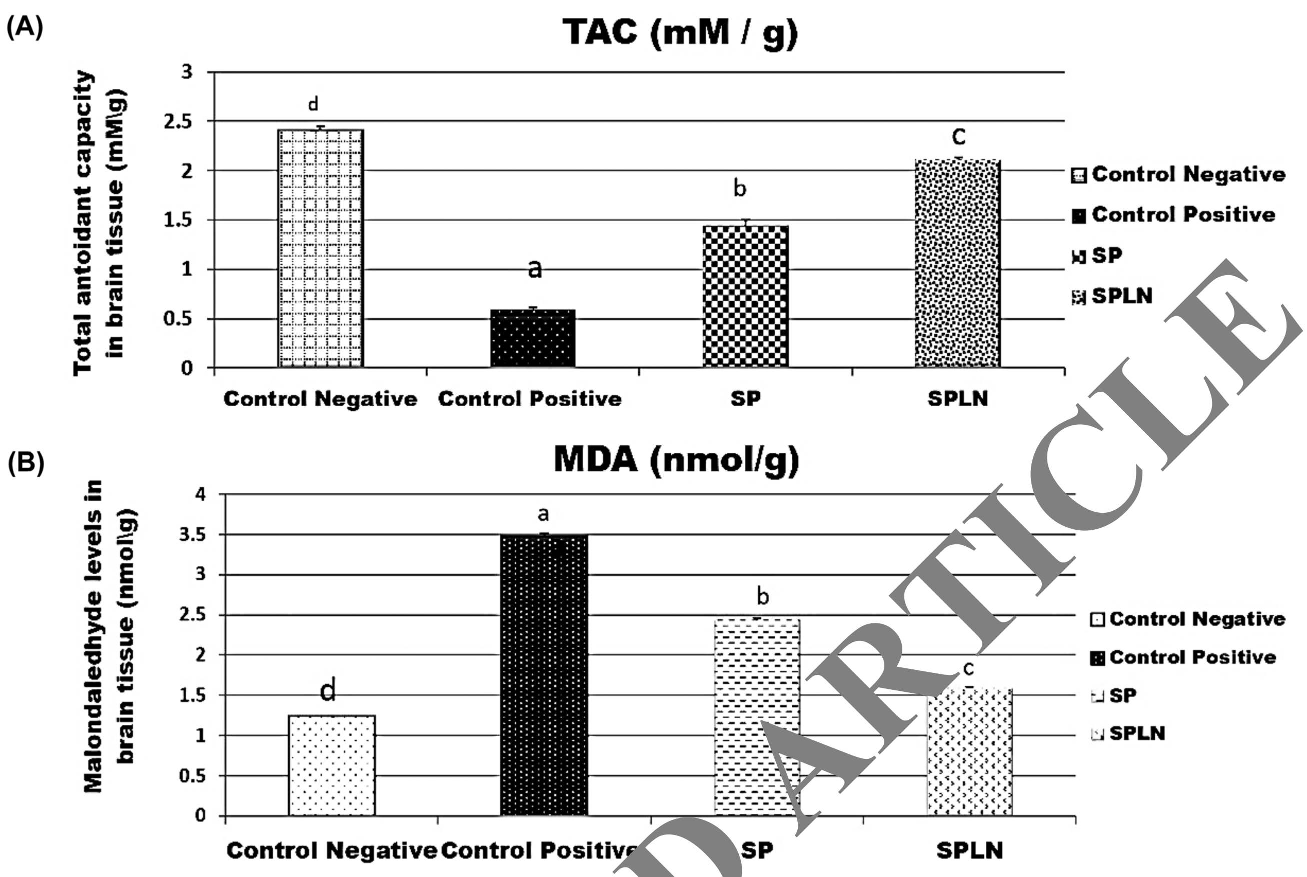

Fig. 4 Effect of SPLN formulation on TAC and MDA levels in rat brain. Results are expressed as means ± SE. The different superscript indicates significant difference at $P < 0.001$

RETRACTED ARTICLE

Results and discussion

Several neuroprotective benefits of SP in various brain regions have been emphasized in numerous in vivo investigations utilizing various animal models [[illegible], 43]. It was speculated that *Spirulina platensis* water extract could be used to treat dementia and thus avoid cognitive decline and memory loss, as well as increasing antioxidant activity. Microglial activation is at least largely responsible for the onset of neurodegeneration, and SP treatment has been demonstrated to be neuroprotective. Together, the results show that SP can protect brain cells from damage by blocking or reversing a variety of inflammatory and oxidative neurotoxic pathways at the cellular and molecular levels. In the present study, we, therefore, explore this substance's ability to protect rats from AD as well as its mode of action.

Preparation and in vitro characterization of Spirulina-loaded niosome formulation

Optimum SP-loaded niosome formulation (SPLN) for in vitro and in vivo characterization was chosen after a literature review [15, 22, 44, 45]. Based on literature reviews, Tween 60 was chosen as a non-ionic surfactant because its long alkyl chains enabled the creation of small niosomes with a high percentage of EE and a stiff vesicular membrane [14, 19, 45, 46]. The high Tween 60's HLB and surface free energy were made for better encapsulation of SP due to its hydrophilicity [14, 17, 19, 45]. The results matched those of Waddad et al. and Nowroozi et al. [17, 45]. Stable niosomal vesicles with high entrapment efficiency are generated in the presence of cholesterol [14, 17, 19, 45]. In addition to lowering the surface free energy, cholesterol also increases the hydrophobicity and stiffness of the bilayer. An increase in %EE and a reduction in particle size were seen in conjunction with a Tween 60/cholesterol ratio of 1:2 [14, 17, 45, 47]. The results matched those of Chaw et al. and Waddad et al. [22, 45]. As a charge inducer, DDP is employed to help ensure that the niosomes are being prepared to have a highly negative zeta potential value, which is good for their stability [19, 45]. According to literature reviews, the optimum formulation included Tween 60, cholesterol, and DDP at the following molar ratios: 1:2:0.1 [14, 17,

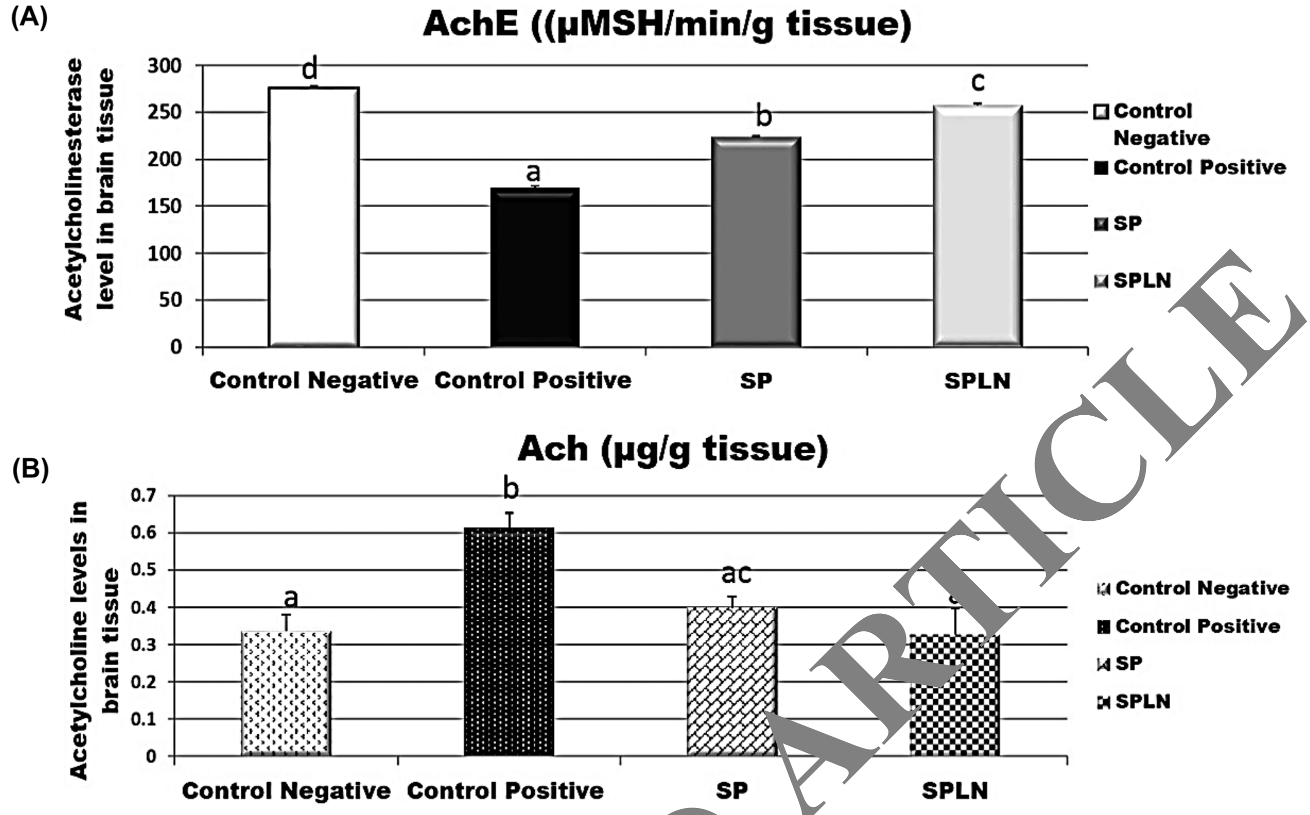

RETRACTED ARTICLE

Fig. 5 Effect of SPLN formulation on AchE and Ach levels in rat brain. Results are expressed as means ± SE. The different superscript indicates a significant difference at $P < 0.05$ and 0.01

19, 45]. Figure 1 displays the morphology of the SPLN formulation, which consisted of round, scattered vesicular structure. The SPLN formulation showed a small particle size of 273.1 ± 8.15 nm and a low PDI of 0.362, indicating a homogeneous niosome, low interfacial tension, and the absence of aggregation tendencies [17, 19, 45]. The SPLN formulation was measured to have a zeta potential of − 5.32 ± 0.67%. Due to electrostatic repulsions between vesicles caused by the negative charge of the SPLN formulation, stable vesicles were created [19, 21, 22, 48].

In vivo characterization of SPLN formulation

Behavioral test

In Fig. 2, a significant ($P < 0.01$) decrease in the discrimination ratio (DR) (Fig. 2A), the discrimination index (DI) (Fig. 2B), and the difference score (DS) (Fig. 2C) was observed in the control positive group compared to the control negative group. Treatments with SP and SPLN formulation improved recognition memory significantly ($P < 0.01$) by increasing DR, DI, and DS when compared to the control positive group. Additionally, the SPLN formulation significantly increased DR ($P < 0.05$) in comparison with SP.

Figure 3 shows that the control positive group saw a statistically significant ($P < 0.01$) reduction in spontaneous alternation behavior (SAP) as compared to the control negative group. When compared to the control positive group, treatment with free SP and SPLN formulation significantly improved the impaired working memory by increasing SAP ($P < 0.01$). Our obtained data clarified that $AlCl_3$ plays a crucial role in AD modeling as it can cross the blood–brain barrier, causing neuron loss, amyloid plaque precipitation, and memory impairment. Consistent with the findings of Singh et al. and Mohamed et al., we discovered that oral treatment of $AlCl_3$ decreased working memory in the y-maze test and short-term memory in the NOR test in AD model rats [49, 50]. Treatment with SP and SPLN formulation improved the impaired recognition memory and spontaneous alternation behavior. Consistent with previous studies by Wang et al. and Imai et al., we found that SP treatment enhanced DI in the novel object test and enhanced spontaneous alternation behavior in the Y-maze in an AD rat model [51, 52]. Additionally, SPLN formulation significantly increased recognition memory and spontaneous alternation behavior in comparison with SP because niosomes improved SP's permeability, localized and stored it in the brain, and then slowly released the medication over time.

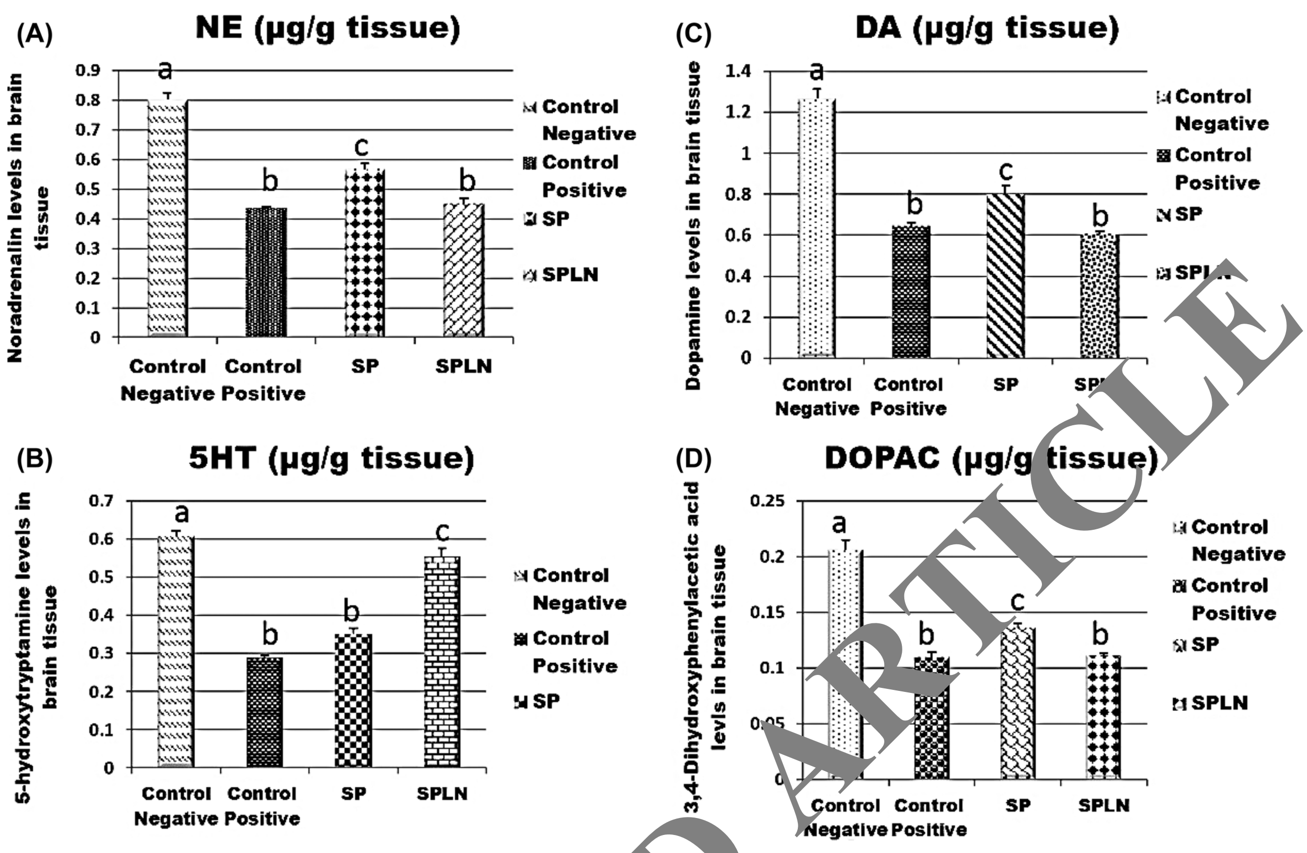

Fig. 6 Effect of SPLN on monoamines levels (NE, 5HT, DA, DOPAC) in rat brain. Results are expressed as means ± SE. The different superscript indicates a significant difference at P < 0.05 and 0.01

RETRACTED ARTICLE

Determination of oxidative stress markers

As shown in Fig. 4, $AlCl_3$ caused significant (*P < 0.01*) oxidative stress in the brain by decreasing the antioxidant marker (TAC, Fig. 4A) while increasing the pro-oxidant marker (MDA, Fig. 4B) compared to the control negative group. Treatments with free SP and SPLN formulation significantly (*P < 0.01*) improved brain oxidative status by decreasing MDA and increasing TAC in comparison with the control positive group. Additionally, SPLN formulation significantly (*P < 0.01*) increased TAC, decreased MDA, and switched the total antioxidant capacity from deviation to control levels in comparison with free SP because niosomes improved SP permeability, localized and stored it in the brain, and then slowly released the medication over time. Our obtained data clarified that $AlCl_3$ as a neurotoxin plays a crucial role in AD modeling, causing oxidative stress by increasing MDA and decreasing TAC. Our results were in line with prior research, which demonstrated a significant rise in brain MDA, while enzymatic antioxidant expression was significantly reduced in the aluminum-treated animals [53, 54]. Treatment with free SP and SPLN formulation alleviated the oxidative stress in the brain. Our results were in line with prior research, which observed that SP has been shown to have antioxidant potential due to the presence of bioactive components such as polysaccharides and carotenoids, which have high antioxidant properties [55]. Exposure of rats to a strength training regimen revealed marked oxidative stress, with elevated MDA and decreased TAC, which was significantly alleviated by SP [56].

Determination of acetylcholinesterase activity, brain monoamines, and acetylcholine levels

The results in Fig. 5 demonstrated that the AchE activity (Fig. 5A) significantly *(P < 0.05)* decreased and the Ach level (Fig. 5B) significantly (*P < 0.05*) increased in the control positive group compared to the control negative group. In comparison to the control positive group, treatments with free SP and SPLN formulation significantly (*P < 0.01*) restored AchE activity and Ach to the normal levels. Our obtained data clarified that $AlCl_3$ plays a crucial role in AD modeling by disturbing brain neurotransmitters. Our results were in line with prior research, which demonstrated a significant decline in AchE activity [50, 57]. Increased Ach level may be attributed to decreased AchE activity, which prevents circulating Ach from being degraded

RETRACTED ARTICLE

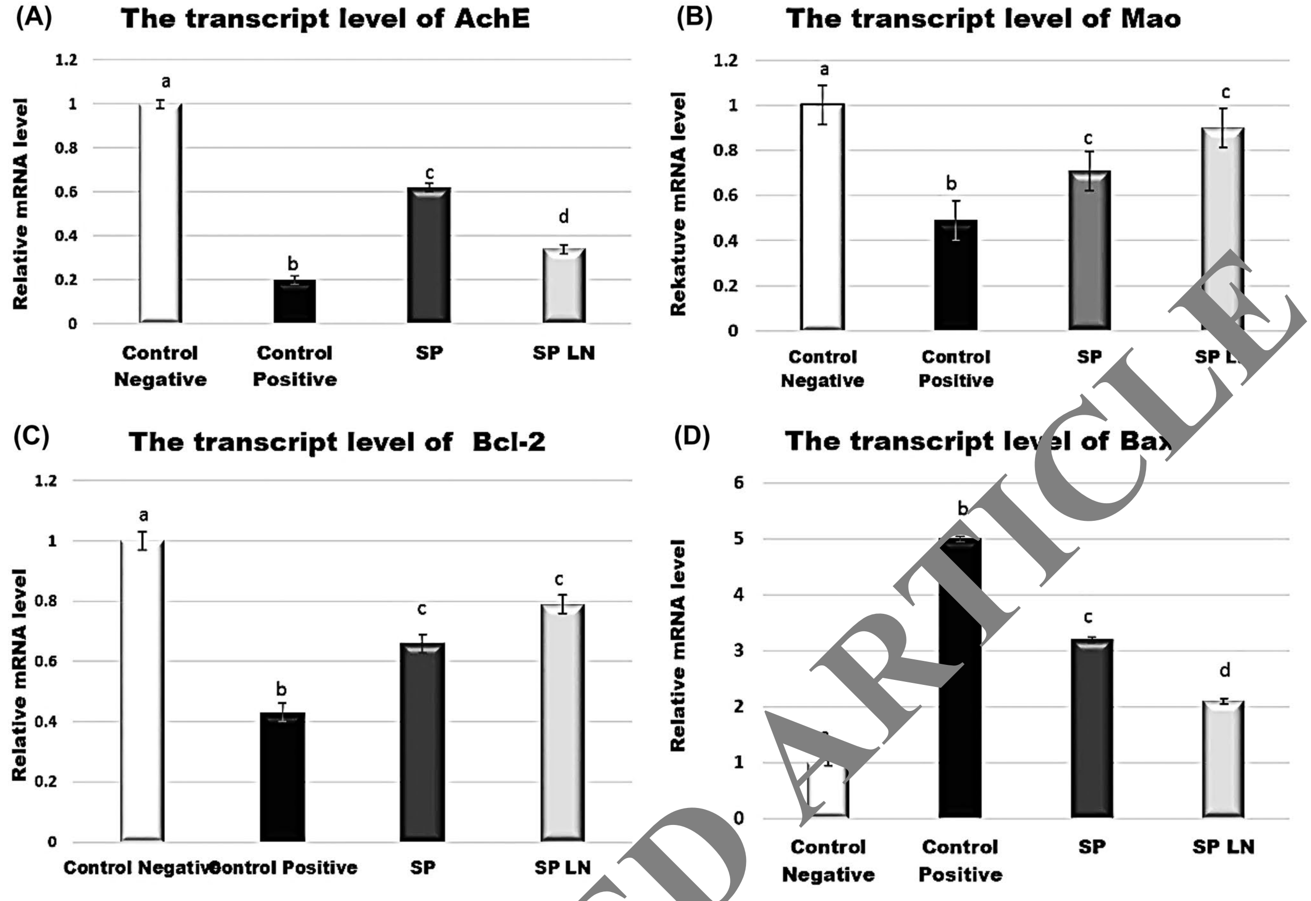

Fig. 7 Effect of SPLN against $AlCl_3$-induced alterations on brain mRNA expression of **A** AchE, **B** Mao, **C** Bcl-2, and **D** Bax. Data are presented as mean ± SE ($n = 6$). The different superscript indicates a significant difference

[58, 59]. Furthermore, $AlCl_3$ resulted in a substantial decline in hippocampal monoaminergic neurotransmitters, mainly dopamine, serotonin, and norepinephrine [6, [illegible]]. Additionally, the antiapoptotic protein (Bcl-2) levels and Mao transcript considerably decreased, and the proapoptotic (Bax) levels increased in the $AlCl_3$-treated group [61]. Moreover, Ibrahim et al. reported that the reduced brain neurotransmitters and downregulation of AchE and Mao resulted from brain neurotoxicity [62]. Aluminum, a metal ion of toxicological relevance, inhibits the activities of Na^+, K^+-activated, and Mg^{2+}-activated adenosine triphosphatase [63]. Compared to the control positive group, treatments with free SP significantly *($P < 0.05$)* increased the reduced AchE activity and upregulated the AchE. The results matched those of Fathy et al. and Galal et al. [64, 65].

Figure 6 shows that the activity of monoamines (NE, 5HT, DA, and DOPAC) in the brain was significantly ($P < 0.05$) lower in the control positive group compared to the control negative group. Treatments with free SP and SPLN formulation restored the brain monoamines' activity significantly *($P < 0.01$)* in comparison to the control positive group. Our obtained data clarified that aluminum neurotoxicity increases the levels of neopterin in the brains of AD patients while also resulting in a decrease in brain neurotransmitters including dopamine, norepinephrine, and serotonin by decreasing the concentrations of tetrahydrobiopterin in the cerebrospinal fluid, which is necessary for the production of such neurotransmitters. The results matched those of Foster et al. [66]. Treatment with SP and SPLN ameliorated the deleterious effect of $AlCl_3$ on brain monoamines. Fathy et al. and Galal et al. recorded a significant increase in brain monoamines levels by SP treatment in rats [64, 65].

Gene expression

Bcl-2 expression has a significant impact on neuronal development during embryogenesis, with Bcl-2 deficient mouse embryos exhibiting significant neuronal development defects [67]. Overexpression of Bcl-2 stimulates DNA repair after DNA damage caused by the oxidative stress. Figure 7 summarizes the gene expression data, revealing an increase in Bax mRNA levels and a decrease in bcl-2, AchE, and Mao mRNA levels in the control positive group compared to the control negative group. These results matched those

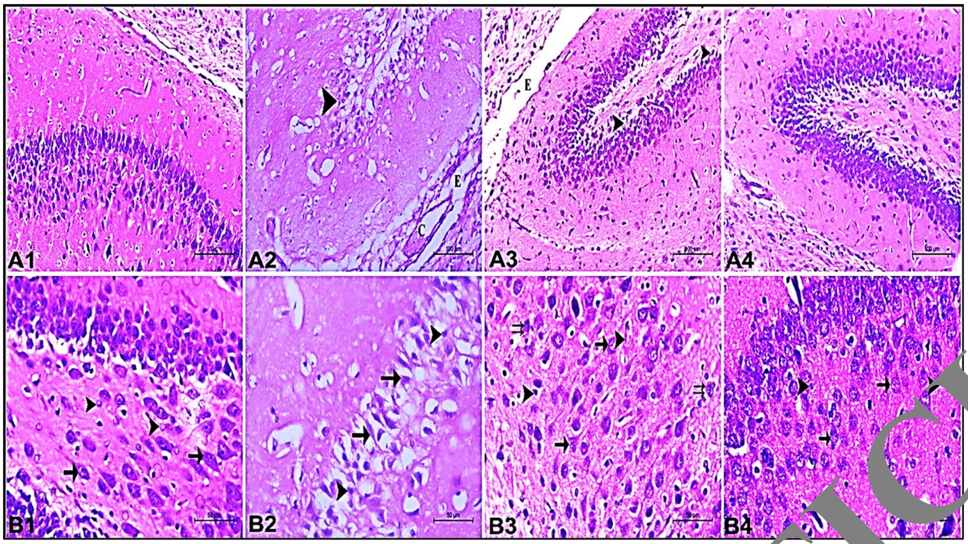

Fig. 8 Histolopathological evaluation (H&E stain). A1: the control negative group showed normal tissue of hippocampus. A2: control positive group showed severe fibrous thickening of the covering of the hippocampus with edema (E) and congested blood vessels (C). The nerve cells and neuroglia showed severe degenerative changes. Accumulation of acidophilic masses (arrowhead). A3: the SP-treated group showed meningeal membrane suffering from edema (E). The cellular layers of hippocampus appeared within normal range with few degenerated neurons and neuroglia cells. A4: the SPLN-treated group showed normal tissue of hippocampus. H&E stain × 200. B1: the control negative group showed the normal architecture of the hippocampus with normal neurons (arrow) as well as normal neuroglia (arrowhead). B2: the control positive group; the hippocampus appeared with few layers. The nerve cells (arrow) and neuroglia cells (arrowhead) showed severe degenerative changes with shrinkage. B3: the SP-treated group showed that the hippocampus was regaining its layers architecture. The majority of nerve cells (arrow) and neuroglial tissue (arrowhead) appeared normal except few cells appeared degenerated (double arrow). B4: the SPLN-treated group revealed normal architecture of the hippocampus containing normal neurons (arrow) and normal neuroglia cells (arrowhead). H&E stain × 400

RETRACTED ARTICLE

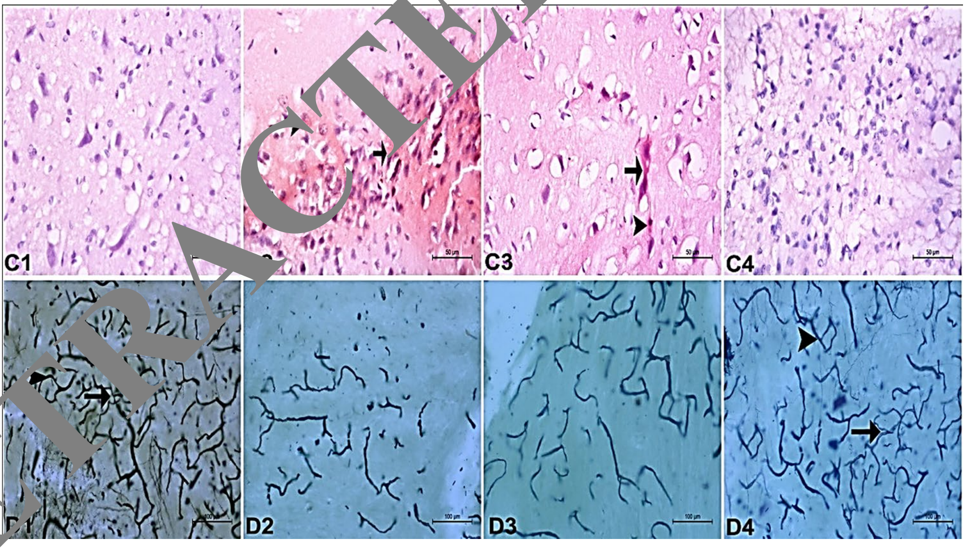

Fig. 9 Histopathological evaluation (Congo red and silver stain). C1: the control negative normal group showed that the hippocampus appeared normal without any foreign materials accumulation. C2: control positive ($AlCl_3$-treated group) showed that a marked deposition of acidophilic amyloid materials in the hippocampus in between (arrow) and/or inside (arrowhead) the neurons. C3: the SP-treated group showed acidophilic materials between the neurons (arrow) and little droplets inside the nerve cells (arrowhead) of the hippocampus. C4: the SPLN-treated group revealed that the hippocampus appeared as a normal group without any amyloid deposition. Congo red stain × 400. D1: the control negative group showed that the tissue of the hippocampus containing highly branched nerve fibers (arrow) with many spots of synapse (arrowhead). D2: control positive ($AlCl_3$-treated group) showed that the nerve fibers of the hippocampus appeared less branched with rare spots of synapse. D3: the SP-treated group revealed that the tissue of hippocampus containing nerve fibers with moderate branching (arrow) with points of synapse (arrowhead). D4: the SPLN-treated group showed the nerve fibers of hippocampus appeared as normal with highly branched neurons (arrow) with many spots of synapse (arrowhead). Silver stain × 200

RETRACTED ARTICLE

of Elmorsy et al. and Ibrahim et al., who found that $AlCl_3$ and other neurotoxins caused a significant downregulation of MAO and AchE as a result of brain neurotoxicity [62, 68]. Treatments with free SP and SPLN formulation ameliorated the injurious effects of the $AlCl_3$. Furthermore, when compared to free SP, SPLN formulation significantly ($P<0.01$) upregulated their gene expression because niosomes improved SP permeability, localized and stored it in the brain, and then slowly released the medication over time. SP treatment significantly increased AchE activity as well as brain monoamine levels in rats, according to Fathy et al. and Galal et al. [64, 65].

Histopathological examination

The normal tissue of the hippocampus was covered by a fine meningeal layer rich with blood vessels. The architecture of the hippocampus appeared normal, with well-demarcated layers of neurons with active nuclei, basophilic cytoplasm, and well-demarcated neuronal processes, as well as normal neuroglia enclosing the nerve cells (Fig. 8, A1 and B1). When stained with Congo red, the hippocampus appeared normal, with no foreign material accumulation. The hippocampus contains highly branched nerve fibers with many spots of synapse (Fig. 9, C1 and D1). The male rats treated with aluminum chloride showed severe tissue damage in the hippocampus in the form of severe fibrous thickening with prominent edema and highly congested blood vessels in the meninges covering the hippocampus. The nerve cells and neuroglial tissue appeared as a few disrupted layers and exhibited severe degenerative changes with massive cell shrinkage as well as marked vacuoles all over the brain tissue (Fig. 8, A2 and B2). Sections of the hippocampus stained with Congo red stains showed deposition of acidophilic amyloid materials in between and/or inside the neurons. The nerve fibers of the hippocampus appeared less branched with rare spots of synapse (Fig. 9, C2 and D2). Buraimoh et al. and Kamel et al. found that rats exposed to oral $AlCl_3$ administration showed neuronal degeneration in various hippocampal areas [69, 70]. Interestingly, Congo red staining confirmed a slight positive reactivity of amyloid plaques that could be seen in the brains of $AlCl_3$-treated rats [6]. The brain tissue in these rats was protected from the harmful action of $AlCl_3$ by administration of SP and SPLN, with the tissue of the hippocampus appearing more or less normal in these groups, respectively (Fig. 8, A3,4 and B3,4; Fig. 9, C3,4 and D3,4), which is supported by Galal et al. and Yousef et al., who discovered that SP treatment improved and reduced neurodegenerative changes in the brain [56, 65].

Novel therapeutics for Alzheimer's disease management, as well as symptom-reducing medications for cognitive decline and neuropsychiatric symptoms, are urgently needed. Our research has looked into the effectiveness of SP and SPLN in alleviating Alzheimer's disease in rats and mice. Surprisingly, SP and SPLN significantly reduced the negative effects of $AlCl_3$ via ameliorating cognitive dysfunction, reducing oxidative stress, restoring brain neurotransmitters, upregulating gene expression, and playing a neuroprotective role in the brain.

Conclusion

Aluminum-induced AD is one of the main risk factors for dementia all over the world. *Spirulina platensis* (SP) has shown anti-inflammatory and antioxidant abilities by reducing oxidative stress, free radicals, and other reactive oxygen species. The purpose of our research was to deliver SP in a controlled and targeted manner using niosomes in order to improve the effectiveness and selectivity of SP as an AD therapy. Different SP-loaded niosome (SPLN) formulations were prepared and evaluated to choose the optimum formulation for further studies such as the efficacy and toxicity of SPLN in comparison to oral SP using the $AlCl_3$-induced AD rat model. Compared with the $AlCl_3$ control group, rats treated with the SPLN formulation showed antioxidant, anti-neuroinflammatory, and cholinergic signaling enhancements. The SPLN formulation enhanced recognition and working memories and restored the reduced brain neurotransmitters. In conclusion, the SPLN formulation could be considered an effective AD therapy.

Acknowledgements All authors extend their sincere thanks, appreciation, and respect to Amoun Vet. Company (AVC) for pharmaceutical drug synthesis, for their assistance with a good and identified well known source of *Spirulina platensis*.

Author contribution All authors contributed in the practical experimental study, design of the experiment, and writing and revision of the manuscript.

Funding Open access funding provided by The Science, Technology & Innovation Funding Authority (STDF) in cooperation with The Egyptian Knowledge Bank (EKB).

Data availability There is no permission for data availability from the other authors.

Declarations

Ethics approval and consent to participate All animal handling, weighting, and treatment were carried out according to the ethical animal's treatment of the Institutional Animal Care and Use Committee (IACUC) of Beni-Suef University, Faculty of Pharmacy with attached approval nomber.

Consent for publication All authors agree about this study publishing.

Conflict of interest The authors declare no competing interests.

Open Access This article is licensed under a Creative Commons Attribution 4.0 International License, which permits use, sharing, adaptation, distribution and reproduction in any medium or format, as long as you give appropriate credit to the original author(s) and the source,

provide a link to the Creative Commons licence, and indicate if changes were made. The images or other third party material in this article are included in the article's Creative Commons licence, unless indicated otherwise in a credit line to the material. If material is not included in the article's Creative Commons licence and your intended use is not permitted by statutory regulation or exceeds the permitted use, you will need to obtain permission directly from the copyright holder. To view a copy of this licence, visit http://creativecommons.org/licenses/by/4.0/.

RETRACTED ARTICLE

References

1. Jack CR Jr, et al. NIA-AA research framework: toward a biological definition of Alzheimer's disease. Alzheimers Dement. 2018;14(4):535–62.
2. Walia V, et al. Correction to: Delineation of neuroprotective effects and possible benefits of antioxidants therapy for the treatment of Alzheimer's diseases by targeting mitochondrial-derived reactive oxygen species: bench to bedside. Mol Neurobiol. 2022;59(1):681–2.
3. Qiu C, Kivipelto M, Von Strauss E. Epidemiology of Alzheimer's disease: occurrence, determinants, and strategies toward intervention. DCNS. 2022.
4. Tahami Monfared AA. et al. Alzheimer's disease: epidemiology and clinical progression. Neurol Ther. 2022:1–17.
5. Livingston G, et al. Dementia prevention, intervention, and care: 2020 report of the Lancet Commission. The Lancet. 2020;396(10248):413–46.
6. Cao Z, et al. Hypericum perforatum extract attenuates behavioral, biochemical, and neurochemical abnormalities in Aluminum chloride-induced Alzheimer's disease rats. Biomed Pharmacother. 2017;91:931–7.
7. Hussien HM, et al. Neuroprotective effect of berberine [illegible]st environmental heavy metals-induced neurotoxicity and [illegible]mer's-like disease in rats. Food Chem Toxicol. 2018;111:432–[illegible]
8. Kawahara M, Kato-Negishi M. Link between aluminu[illegible] the pathogenesis of Alzheimer's disease: the integration of t[illegible]e alu[illegible]um and amyloid cascade hypotheses. Int J Alzheimer'[illegible] Dis. 2011.
9. Sorrenti V, et al. Spirulina microalgae and brai[illegible] health: a scoping review of experimental and clinical evidence. Mar [illegible]ugs. 202[illegible];19(6):293.
10. Brito AdF, et al. Spirulina platensis preven[illegible]idative stress and inflammation promoted by strength[illegible]ining in rats: dose-response relation study. Sci Rep. 2020;10(1)[illegible]1–[illegible]
11. Ibrahim F, et al. Manganes[illegible]induced neurotoxicity and the potential protective effects of lipo[illegible] acid an[illegible] Spirulina platensis. Toxicol Mech Methods. 20[illegible]30[illegible]–507.
12. Sibiya T, Ghazi T, Chutur[illegible]n A. The potential of Spirulina platensis to amelio[illegible] the advers[illegible] effects of highly active antiretroviral therapy ([illegible]AAR[illegible] Nutrients. 2022;14(15):3076.
13. Alheeti [illegible]N, et al. Th[illegible] potential protective effect of Spirulina nano[illegible]ticles against ehrlich solid tumor bearing mice induced liver to[illegible]y, tum[illegible]r markers, DNA fragmentation, oxidative stress [illegible]monoo[illegible]genase variations. 2021.
1[illegible]. Ma[illegible]osroi A, et al. Characterization of vesicles prepared with vari[illegible]onic surfactants mixed with cholesterol. Colloids Surf, B[illegible]003;30(1–2):129–38.
15. Ka[illegible] KM, et al. Niosome: a future of targeted drug delivery systems. Journal of advanced pharmaceutical technology & research. 2010;1(4):374.
16. Arora D, et al. QbD-based rivastigmine tartrate loaded solid lipid nanoparticles for enhanced intranasal delivery to the brain for Alzheimer's therapeutics. Front Aging Neurosci. 2022:869.
17. Nowroozi F, et al. Effect of surfactant type, cholesterol content and various downsizing methods on the particle size of niosomes. Iranian Journal of Pharmaceutical Research: IJPR. 2018;17(Suppl2):1.
18. Haddadian A, et al. Niosomes-loaded selenium nanoparticles as a new approach for enhanced antibacterial, anti-biofilm, and anticancer activities. Sci Rep. 2022;12(1):1–16.
19. Bnyan R, et al. Surfactant effects on lipid-based vesicles properties. J Pharm Sci. 2018;107(5):1237–46.
20. Maurizi L, et al. Effect of ciprofloxacin-loaded niosomes on Escherichia coli and Staphylococcus aureus biofilm formation. Pharmaceutics. 2022;14(12):2662.
21. Gamal A, et al. Improving the antitumor activity and [illegible]ioavailability of sonidegib for the treatment of skin cancer. P[illegible]ma[illegible]eutics. 2021;13(10):1560.
22. Chaw CS, Ah Kim KY. Effect of formulati[illegible]n composi[illegible]s on niosomal preparations. Pharm Dev Techno[illegible]13;18(3)[illegible]667–672.
23. Ahmad Rather M, et al. Asiatic acid a[illegible]nuate[illegible]lumi[illegible]num chloride-induced tau pathology, oxida[illegible]ve stress an[illegible]apoptosis via AKT/GSK-3β signaling pathway [illegible] Wistar ra[illegible]ts. Neurotox Res. 2019;35(4):955–68.
24. Auti ST, Kulkarni YA. Neuro[illegible]tective [illegible]ffect of cardamom oil against aluminum indu[illegible]ed neur[illegible]xicity in rats. Front Neurol. 2019;10:399.
25. Khalil SR, et al. [illegible]piru[illegible] platensis attenuates the associated neurobeh[illegible]oral and [illegible]flammatory response impairments in ra[illegible] expo[illegible]ed to lead acetate. Ecotoxicol Environ Saf. 2018;157:25[illegible]5.
26. Bevins RA, Bes[illegible]r J. Object recognition in rats and mice: a one-trial n[illegible]matching[illegible]to-sample learning task to study "recognition memor[illegible]otoc. 2006;1(3):1306–11.
27. Leger [illegible], et al. Object recognition test in mice. Nat Protoc. 2013;8(1[illegible]):2531–7.
28. [illegible]im S, et al. Ginger improves cognitive function via NGF-induced [illegible]K/CREB activation in the hippocampus of the mouse. J Nutr [illegible]iochem. 2014;25(10):1058–65.
29. Roghani M. The effect of Nigella sativa on learning and memory in male diabetic rats. Basic and Clinical Neuroscience. 2009;1(1):32.
30. Wall P, et al. Infralimbic D1 receptor agonist effects on spontaneous novelty exploration and anxiety-like defensive responding in CD-1 mice. Behav Brain Res. 2004;152(1):67–79.
31. Baluchnejadmojarad T, et al. The effect of alpha-lipoic acid on learning and memory deficit in a rat model of temporal lobe epilepsy. Basic and Clinical Neuroscience. 2012;3(3):58.
32. Rasoulijazi H, et al. The beneficial effect of (-)-epigallocatechin-3-gallate in an experimental model of Alzheimer's disease in rat: a behavioral analysis. 2007.
33. Ragy MM. Effect of exposure` and withdrawal of 900-MHz-electromagnetic waves on brain, kidney and liver oxidative stress and some biochemical parameters in male rats. Electromagn Biol Med. 2015;34(4):279–84.
34. Raghuveeran C. Reversed phase ion-pair chromatographic separation of acetyl choline and choline. J Liq Chromatogr. 1985;8(3):537–44.
35. Arafa A, et al. Phylogenetic analysis of hemagglutinin and neuraminidase genes of highly pathogenic avian influenza H5N1 Egyptian strains isolated from 2006 to 2008 indicates heterogeneity with multiple distinct sublineages. Avian Dis. 2010;54(s1):345–9.
36. Cazenave S, Chapoulie R, Villeneuve G. Cathodoluminescence of synthetic and natural calcite: the effects of manganese and iron on orange emission. Mineral Petrol. 2003;78(3):243–53.
37. Ko JH, et al. Cloning of large-conductance Ca(2+)-activated K(+) channel alpha-subunits in mouse cardiomyocytes. Biochem Biophys Res Commun. 2009;389(1):74–9.
38. Ahmed WMS, et al. Neuromodulatory effect of cinnamon oil on behavioural disturbance, CYP1A1, iNOStranscripts and

neurochemical alterations induced by deltamethrin in rat brain. Ecotoxicol Environ Saf. 2021;209:111820.
39. Bancroft JD, Gamble M. Theory and practice of histological techniques. Elsevier health sciences. 2008.
40. Cheng Y, et al. Proteomic identification of calcium-binding chaperone calreticulin as a potential mediator for the neuroprotective and neuritogenic activities of fruit-derived glycoside amygdalin. J Nutr Biochem. 2015;26(2):146–54.
41. Lee SW. Methods for testing statistical differences between groups in medical research: statistical standard and guideline of Life Cycle Committee. Life Cycle. 2022;2.
42. Hwang J-H, et al. Spirulina prevents memory dysfunction, reduces oxidative stress damage and augments antioxidant activity in senescence-accelerated mice. J Nutr Sci Vitaminol. 2011;57(2):186–91.
43. Pabon MM, et al. A Spirulina-enhanced diet provides neuroprotection in an α-synuclein model of Parkinson's disease. 2012.
44. Chauhan MK, Bhatt N. Bioavailability enhancement of polymyxin B with novel drug delivery: development and optimization using quality-by-design approach. J Pharm Sci. 2019;108(4):1521–8.
45. Waddad AY, et al. Formulation, characterization and pharmacokinetics of Morin hydrate niosomes prepared from various non-ionic surfactants. Int J Pharm. 2013;456(2):446–58.
46. Abdelbary G, El-Gendy N. Niosome-encapsulated gentamicin for ophthalmic controlled delivery. AAPS PharmSciTech. 2008;9(3):740–7.
47. Homaei M. Preparation and characterization of giant niosomes. 2016.
48. Shuwaili AHA, Rasool BKA, Abdulrasool AA. Optimization of elastic transfersomes formulations for transdermal delivery of pentoxifylline. Eur J Pharm Biopharm. 2016;102:101–14.
49. Mohamed AB, Mohamed AZ, Aly S. Effect of thymoquinone against aluminum chloride-induced Alzheimer-like model in rats: a neurophysiological and behavioral study. Med J Cairo Univ. 2020;88:355–365.
50. Singh NA, et al. EGCG nanoparticles attenuate aluminum chloride induced neurobehavioral deficits, beta amyloid and tau pathology in a rat model of Alzheimer's disease. Frontiers in aging neuroscience. 2018;10:244.
51. Imai Y, et al. Nutrigenomic studies on the ameliorative effect of enzyme-digested phycocyanin in Alzheimer's disease model mice. Nutrients. 2021;13(12):4431.
52. Wang P, et al. Amelioration of cognitive deficits by Spirulina platensis in L-methionine-induced rat model of vascular dementia. Pharmacogn Mag. 2020;16(68):13[illegible]
53. Doungue HT, Kengne APN, Kuate D. Neuroprotective effect and antioxidant activity of Passiflora edulis fruit flavonoid fraction, aqueous extract, and juice in aluminum chloride-induced Alzheimer's disease rats. Nutr[illegible] 20[illegible](1):1–12.
54. Sadek KM, Lebda MA, A[illegible]zed TK. The possible neuroprotective effects of melatonin in aluminum chloride-induced neurotoxicity via antioxidant pathway and Nrf2 signaling apart from metal chelation. Environ Sci Pollut Res. 2019;26(9):9174–83.
55. Han P, et al. Anti-oxidation properties and therapeutic potentials of spirulina. Algal Res. 2021;55:102240.
56. Yousef MI, et al. Neuroprotective potential of Spirulina platensis against aluminium chloride-induced neural degeneration. Curr Top Nutraceutical Res. 2020;18(4).
57. Exley C, Vickers T. Elevated brain aluminium and early onset Alzheimer's disease in an individual occupationally exposed to aluminium: a case report. J Med Case Reports. 2014;8(1):1–3.
58. Gulya K, Rakonczay Z, Kasa P. Cholinotoxic effects of aluminum in rat brain. J Neurochem. 1990;54(3):1020–6.
59. Julka D, Sandhir R, Gill KD. Altered cholinergic metabolism in rat CNS following aluminum exposure: implications on learning performance. J Neurochem. 1995;65(5):2157–64.
60. Abbas F, et al. Celastrol and thymoquinone alleviate aluminum chloride-induced neurotoxicity: Behavioral psychomotor performance, neurotransmitter level, oxidative-inflammatory markers, and BDNF expression in rat brain. Biomed Pharmacother. 2022;151:113072.
61. Mesole SB, et al. Apoptotic inducement of neuronal cells by aluminium chloride and the neuroprotective effect of eugenol in wistar rats. Oxid Med Cell Longev. [illegible]20.
62. Ibrahim AE-M, et al. Single or combined exposure to chlorpyrifos and cypermethrin provoke oxidative stress and downregulation in monoamine oxidase and acetylcholinesterase gene expression of the rat's brain. Environ Sci Pollut Res. 2020;27(11):12692–703.
63. Patočka J, Bajgar J. Aluminium activation and inhibition of human brain acetylcholinesterase in vitro. Inorg Chim Acta. 1987;135(2):[illegible].
64. Fathy SM, Essa [illegible]. Influence of Spirulina platensis exudates on the endocrine and nervous systems of a mammalian model. Asian Pac J Trop Biomed. 2015;5(6):451–7.
65. Galal MK, et al. Modulation of caspase-3 gene expression and protective effects of garlic and spirulina against CNS neurotoxicity induced by lead exposure in male rats. Neurotoxicology. 2019;72:15–28.
66. Foster HD. How aluminum causes Alzheimer's disease: the implications for prevention and treatment of Foster's multiple antagonist hypothesis. Journal of Orthomolecular Medicine. 2000;15(1):21–51.
67. Abdelghany AK, et al. Neuroprotective role of medicinal plant extracts evaluated in a scopolamine-induced rat model of Alzheimer's disease. Biomarkers : biochemical indicators of exposure, response, and susceptibility to chemicals. 2022;27(8):773–83.
68. Elmorsy E, et al. The protective effect of Indian Catechu methanolic extract against aluminum chloride-induced neurotoxicity, a rodent model of Alzheimer's disease. Heliyon. 2021;7(2):e06269.
69. Buraimoh A, et al. Effects of oral administration of aluminium chloride on the histology of the hippocampus of wistar rats. Curr Res J Biol Sci. 2011;3(5):509–15.
70. Kamel ES, Mostafa N. Effect of aluminum chloride on the hippocampus of adult rats and the possible protective role of Nigella sativa: a histological and immunohistochemical study. Egypt J Histol. 2013;36(2):505–13.

Publisher's Note Springer Nature remains neutral with regard to jurisdictional claims in published maps and institutional affiliations.

RETRACTED ARTICLE
